# Integrated Cascade Biorefinery Processes to Transform Woody Biomass Into Phenolic Monomers and Carbon Quantum Dots

**DOI:** 10.3389/fbioe.2021.803138

**Published:** 2021-12-23

**Authors:** Xue Chen, Jiubin Zhu, Wenlu Song, Ling-Ping Xiao

**Affiliations:** ^1^ Department of Life Science and Engineering, Jining University, Jining, China; ^2^ Liaoning Key Lab of Lignocellulose Chemistry and BioMaterials, Liaoning Collaborative Innovation Center for Lignocellulosic Biorefinery, College of Light Industry and Chemical Engineering, Dalian Polytechnic University, Dalian, China; ^3^ Guangxi Key Laboratory of Clean Pulp and Papermaking and Pollution Control, College of Light Industry and Food Engineering, Guangxi University, Nanning, China

**Keywords:** phenolic monomer, reductive catalytic fractionation, carbon quantum dots, fluorescent sensing, hydrogenolysis

## Abstract

A novel cascade biorefinery strategy toward phenolic monomers and carbon quantum dots (CQDs) is proposed here *via* coupling catalytic hydrogenolysis and hydrothermal treatment. Birch wood was first treated with catalytic hydrogenolysis to afford a high yield of monomeric phenols (44.6 wt%), in which 4-propanol guaiacol (10.2 wt%) and 4-propanol syringol (29.7 wt%) were identified as the two major phenolic products with 89% selectivity. An available carbohydrate pulp retaining 82.4% cellulose and 71.6% hemicellulose was also obtained simultaneously, which was further used for the synthesis of CQDs by a one-step hydrothermal process. The as-prepared CQDs exhibited excellent selectivity and detection limits for several heavy metal cations, especially for Fe^3+^ ions in an aqueous solution. Those cost-efficient CQDs showed great potential in fluorescent sensor *in situ* environmental analyses. These findings provide a promising path toward developing high-performance sensors on environmental monitoring and a new route for the high value-added utilization of lignocellulosic biomass.

## Introduction

Lignocellulosic biomass, sustainable and high-energy content stored in the biosphere, is regarded as a promising feedstock for the production of sustainable materials and chemicals (Li et al., 2020). Lignin as the largest naturally occurring aromatic/phenolic compound accounts for 15–40% of lignocellulosic biomass (Ragauskas et al., 2014). Depolymerizing lignin into monomeric phenols would be a vital starting point for improving the resulting lignin fraction utilization (Sun et al., 2020). However, the current lignocellulosic biorefinery focuses on carbohydrate (cellulose and hemicellulose) valorization, including sulfite, kraft, and organosolv pretreated processes. During those processes, stable C–C linkages would inevitably be formed in the separated lignin samples, which predominantly affect the depolymerization of lignin into monomeric phenols (Renders et al., 2017). Therefore, the harnessing of lignin in a practical manner is still a major challenge.

Numerous efforts have been made to transfer the lignin into low–molecular weight aromatics, which would compete directly against existing chemicals derived from petroleum. The reductive catalytic fractionation (RCF) (also denominated as “lignin-first” strategy) can convert protolignin into monomeric phenols with a high yield, while keeping carbohydrates with very high retention in the solid residues (Renders et al., 2017). The biorefinery approach of the RCF would achieve high yields of overall products from all three biopolymers (cellulose, hemicellulose, and lignin) *via* a combined process, which remains enormous potential to advance biorefinery technology and economy (Schutyser et al., 2018; Wang et al., 2020c). Until now, heterogeneous metal catalysts, including noble [Pd (Zhang et al., 2019), Ru (Van den Bosch et al., 2015a; Li and Song, 2019), Pt (Xu et al., 2012), and Rh (Liu Y. et al., 2019)] or non-noble [Ni (Wang et al., 2019), Mo (Xiao et al., 2017; Sun et al., 2019), and Cu (Sun et al., 2018)] metals and bimetallic catalysts [NiMo (Wang Y.-Y. et al., 2016), MoZn (Wang et al., 2018), and Zn/Pd/C (Parsell et al., 2015)], were usually employed for lignin depolymerization. During RCF, the utilization of metal catalysts can motivate the cleavage of ether C–O bonds and avoid the formation of new stable C–C bonds of protolignin (Renders et al., 2017). Generally, the selective control of phenolic monomers with different end-chains at the para position has been achieved through the selection of catalysts, resulting in the formation of propyl- (Parsell et al., 2015), propenyl- (Galkin and Samec, 2014), propanol- (Van den Bosch et al., 2015a), ethyl-, and allyl ether (Xiao et al., 2017; Sun et al., 2019)-substituted phenols. The solid residues of the RCF process are mainly composed of almost all the hemicellulose and cellulose components. Although high yields of phenolic monomers were obtained during RCF, only few studies have focused on the further application of the carbohydrate in the solid fraction. Zhang et al. (2019) reported that cellulose and hemicellulose components in solid pulp could be enzymatically hydrolyzed into monosaccharides with 90% yield of glucose and 85% yield of xylose, respectively. Van den Bosch et al. (2015b) studied the chemocatalytic conversion of hemicellulose residues into pentitols and cellulose into hexitols by using a Ru/C catalyst. In our previous work, we reported that the solid pulp obtained from Pd-catalyzed *Eucalyptus* wood could be efficiently converted into levulinic acid and furfural using FeCl_3_ catalysis process (Chen et al., 2020). Therefore, the exploration of high-value added solid pulp utilization would advance the transformation of all components of biomass.

Carbon quantum dots (CQDs), a novel fluorescent carbon material, have attracted tremendous attention because of their unique physicochemical properties and potential applications. CQDs exhibit a large variety of merits, such as stable fluorescence intensity, good water solubility, excellent biocompatibility, and high photostability (Lim et al., 2015). CQDs demonstrate great potential for various applications in fields of cellular imaging, biosensors, photocatalysis, and light-emitting diodes (Qiao et al., 2019). Given the importance of CQDs, the synthesis of CQDs from a wide range of carbon sources has been explored by hydrothermal treatments, including chitosan (Liang et al., 2016), polyaniline (Qin et al., 2012), sodium citrate (Liu Y. et al., 2017), and carbon paper (Devi et al., 2018). Recently, biomass materials are considered as the potential substitutional feedstocks due to their green, renewable, and available nature. Therefore, the solid carbohydrate pulp, which is generated from RCF of biomass, would be a promising candidate for CQD production.

We have previously reported the RCF of lignocellulosic biomass by using Ru (Li and Song, 2019), Mo (Xiao et al., 2017; Sun et al., 2019), Pd (Zhang et al., 2019; Wang et al., 2020b; Chen et al., 2020), and Ni catalysts (Liu X. et al., 2019; Wang et al., 2019). The further exploration of the transformation of biomass based on the RCF process remains desirable. In the present work, birch wood first underwent catalytic hydrogenolysis to afford a high yield of monomeric phenols. Subsequently, the solid carbohydrate pulp was further treated using a hydrothermal process for the synthesis of CQDs. Finally, the method introduced in this study delivers the enlightening insights to generate value-added chemicals and CQDs from birch wood.

## Materials and Methods

### Materials

Birch wood (*Betula alnoides*) was harvested in Yunnan Province, southwest of China, which consisted of cellulose (38.6%), hemicellulose (22.5%), and lignin (20.1%). The dried birch was grounded to obtain 40–60 mesh wood particles, and the birch powder was extracted with toluene–ethanol (2:1, v/v) for 6 h to remove extractives. The dewaxed birch powder was dried at 60°C. Pd/C catalyst, AlCl_3_, CeH_12_N_3_O_15_, CaCl_2_, FeCl_2_·4H_2_O, CoCl_2_·6H_2_O, FeCl_3_·6H_2_O, CuCl_2_·2H_2_O, KCl, MgCl_2_, and ZnCl_2_ were purchased from Energy Chemical (China).

### Experiment Procedure

The dewaxed birch (1.0 g), methanol (40 ml), and Pd/C (100 mg, 10 wt%) were added into a Parr reactor. The reactor was purged with N_2_ and pressurized with 3 MPa H_2_. The mixture was treated at 240°C for 4 h. After reaction, the mixture was immediately cooled by ice water and then filtered with a Buchner funnel. The insoluble fractions were thoroughly washed with methanol and freeze-dried for further experiment. The soluble fraction containing phenolic compounds was collected for further analysis.

The insoluble fractions consisted of carbohydrate pulp and Pd/C, which could be separated according to 300 mesh screening. Then, the carbohydrate pulp was used to fabricate CQDs. In brief, 0.5 g of solid pulp and 10 ml of water were mixed. The mixture was then ultrasonicated for 30 min to obtain a well-dispersed solution. The mixture was heated to 200°C and maintained for 5 h. Upon completion of the treatment, the mixture was cooled and filtered to obtain the supernatant. The brown supernatant containing water-soluble CQDs was dialyzed in a dialysis membrane (MWCO of 500 Da) for 72 h. The purified CQDs were freeze-dried for further characterization.

### Analysis of Lignin Products

The soluble fraction was extracted with CH_2_Cl_2_–H_2_O. Subsequently, CH_2_Cl_2_ in the organic phase was removed, giving the brown “lignin oil.” The analyses of lignin oil including GC-MS, GC, and gel permeation chromatography (GPC) were described earlier (Chen et al., 2020). The monomeric phenol yields and carbohydrate retention were also calculated based on the equations as described by Zhang et al. (2019).

### Characterization of CQDs

The characterization of CQDs including atomic force microscopy (AFM) image, transmission electron microscopy (TEM) image, X-ray photoelectron spectroscopy (XPS), Fourier transform infrared (FT-IR) spectra, X-ray diffraction (XRD) analysis, and ultraviolet–visible (UV/Vis) spectra were extensively detected as described earlier (Liu et al., 2020).

Quinine sulfate with a 54% quantum yield (QY) in 0.1 M H_2_SO_4_ was used as the standard sample for the calculation of the QY (Sahu et al., 2012). The QY of CQDs was calculated using the equation as described by Liu et al. (2020).

### Fluorescent Detection of Metal Ions

The CQD solution was initially prepared with a concentration of 0.02 mg/ml. Various metal ions including AlCl_3_, CeH_12_N_3_O_15_, CaCl_2_, FeCl_2_·4H_2_O, CoCl_2_·6H_2_O, FeCl_3_·6H_2_O, CuCl_2_·2H_2_O, KCl, MgCl_2_, and ZnCl_2_ were employed for the selectivity study. The concentration of metal ion salt solutions was 2,000 μM, and the pH was adjusted to 3. To evaluate the sensitivity toward Fe^3+^, different concentrations of Fe^3+^ (0–400 μM) were added into the prepared CQD solution for photoluminescence spectra measurement. During the process, 2 ml metal ion salt solution was poured into 2 ml CQD solution and incubated for 5 min. The fluorescent intensity of the CQDs was detected under excitation wavelength at 350 nm, which was named as I. The fluorescent intensity for CQD solution was defined as I_0_.

## Results and Discussion

### Phenolic Monomers From RCF of Birch

**FIGURE 1:**
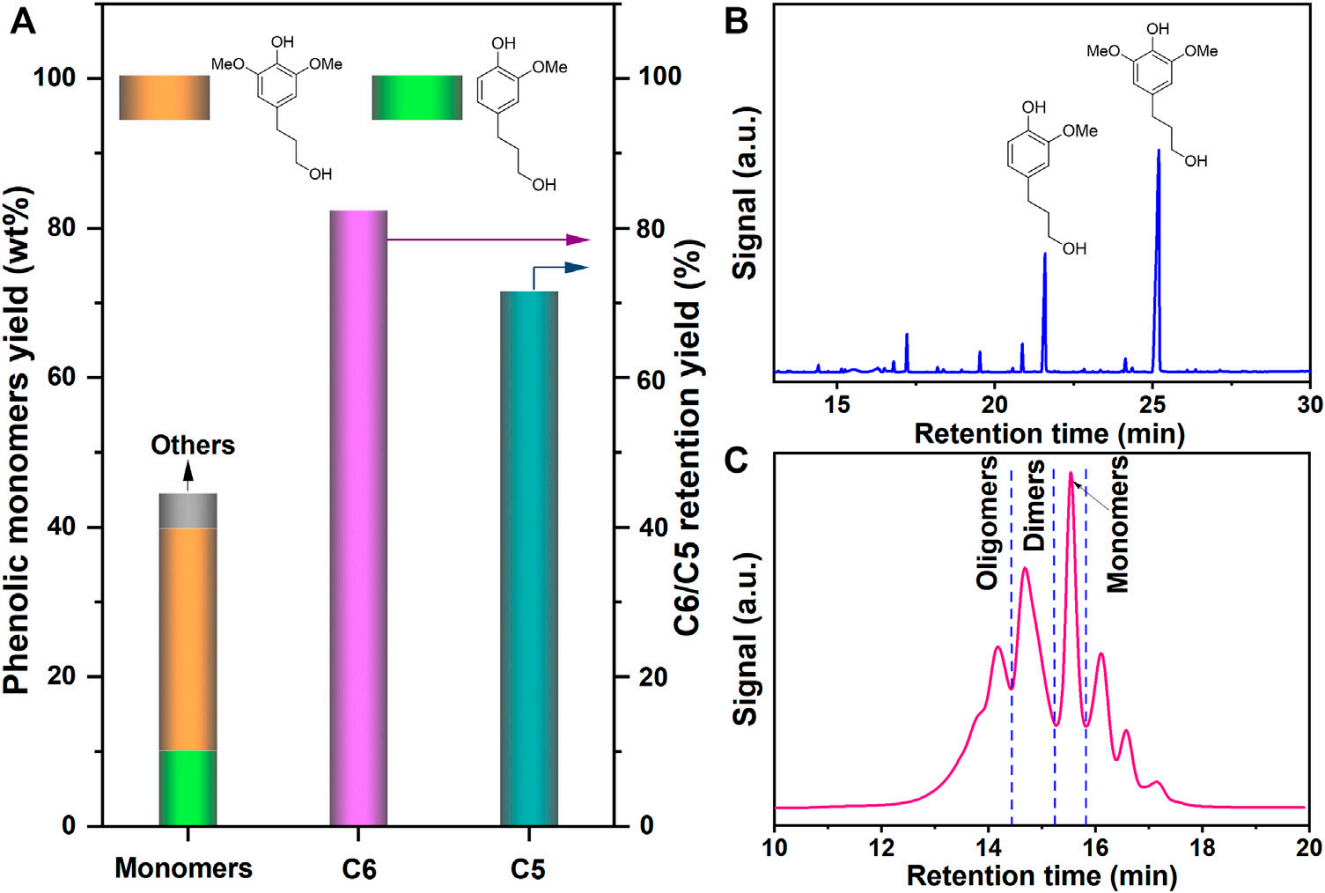
**(A)** Phenolic monomer yield, sugar retention, **(B)** gas chromatogram, and **(C)** molecular weight distribution after RCF of birch.

A wide variety of heterogeneous metal catalysts were used for catalytic hydrogenolysis of lignocellulosic biomass. Considering the phenolic monomer yield, carbohydrate pulp retention, and catalytic ability, Pd/C catalyst was chosen for the RCF of birch (Zhang et al., 2019). The birch wood was treated with Pd/C at 240°C for 4 h using 3 MPa H_2_ in methanol, giving a soluble fraction and solid carbohydrate residues. The soluble fraction was extracted with CH_2_Cl_2_–H_2_O. The brown soluble oily products in the organic phase contained phenolic monomers, dimers, and oligomers, while the aqueous phase mainly composed of sugars. The phenolic monomer yield, the molecular weight of the lignin oily products, and the carbohydrate retention in the solid residues were systematically analyzed. The molecular weight of lignin oil was 440 g mol^−1^, which was significantly decreased as compared to that of the isolated cellulolytic enzyme lignin (CEL, 18,300 g mol^−1^) (Wen et al., 2013) and milled wood lignin (MWL, 10,860 g/mol) from birch (Zhou et al., 2012). As shown in Figure 1A, it was also shown that the total yields of monomeric phenols reached 44.6 wt%. The phenolic monomer yield from the catalytic hydrogenolysis of birch using the Pd/C catalyst was comparable to other reported Pd/C-catalyzed hydrogenolysis of biomass. Zhang et al. (2019) investigated the RCF of bamboo in the presence of Pd/C catalyst, and 41.7 wt% of phenolic monomer yield was obtained. Parsell et al. (2015) reported a bimetallic Zn/Pd/C catalyst that converts lignin in intact lignocellulosic biomass directly to two methoxyphenol products (2-methoxy-4-propylphenol and 2,6-dimethoxy-4-propylphenol), which resulted in 40–54% of the available lignin. The catalytic conversion of lignin in birch to aromatic products by using the Pd/C catalyst was also explored, with a total yield of aromatic products of 49% (Galkin and Samec, 2014). Notably, analysis of the crude bio-oil by GC and GC-MS as illustrated in Figure 1B and Supplementary Table S2 indicated that 4-propanol guaiacol (10.2 wt%) and 4-propanol syringol (29.7 wt%) were the major products of Pd/C-catalyzed hydrogenolysis of birch wood. This result was further supported by the 2D HSQC NMR spectra of the lignin oil, where newly dominant cross-peaks corresponding to propanol moieties were clearly observed, as we previously reported (Chen et al., 2020). Moreover, the selectivity of those two major aromatic phenols reached 89% based on a total monomers yield of 44.6 wt%. Additionally, the direct RCF of birch also afforded the carbohydrate pulp remained as a solid residue with 82.4 wt% C6 and 71.6 wt% C5 retention according to the chemical composition analysis (Figure 1A; Supplementary Table S1), which makes it a promising candidate for the co-production of CQDs.

### Structural Characterization of the CQDs

CQDs were synthesized by a facile hydrothermal treatment using carbohydrate pulp as the carbon source. The effect of reaction time on the QY of CQDs was investigated at 200°C (Supplementary Figure S3). The QY of CQDs enhanced with the extending of retention time from 4 to 8 h, while it decreased with the further prolonging time. Hence, the optimal reaction condition for the synthesis of CQDs was established as 200°C and 5 h, from which the QY of CQDs was calculated to be 21.7%.

**FIGURE 2:**
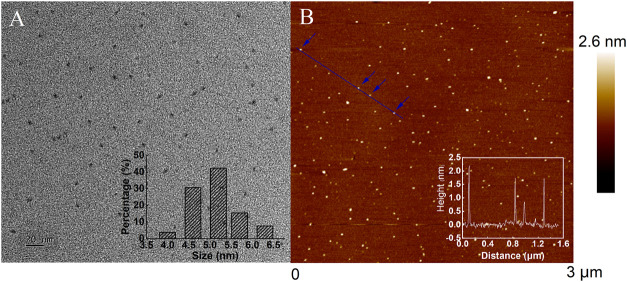
**(A)** TEM and **(B)** AFM images of the CQDs.

The microstructure and morphology of CQDs were characterized by TEM, which illustrated that CQDs had spherical morphologies and were uniformly dispersed in aqueous solutions. As shown in Figure 2A, the particle size of CQDs was distributed in the range of 4.0–6.3 nm, and the average diameter of CQDs was calculated to be 5.24 nm. Figure 2B demonstrates the topographic morphology of the CQDs, and the inset displays an AFM trace between four particles. The AFM image indicates that the heights of the prepared CQDs were in the range of 1–3.6 nm.

**FIGURE 3:**
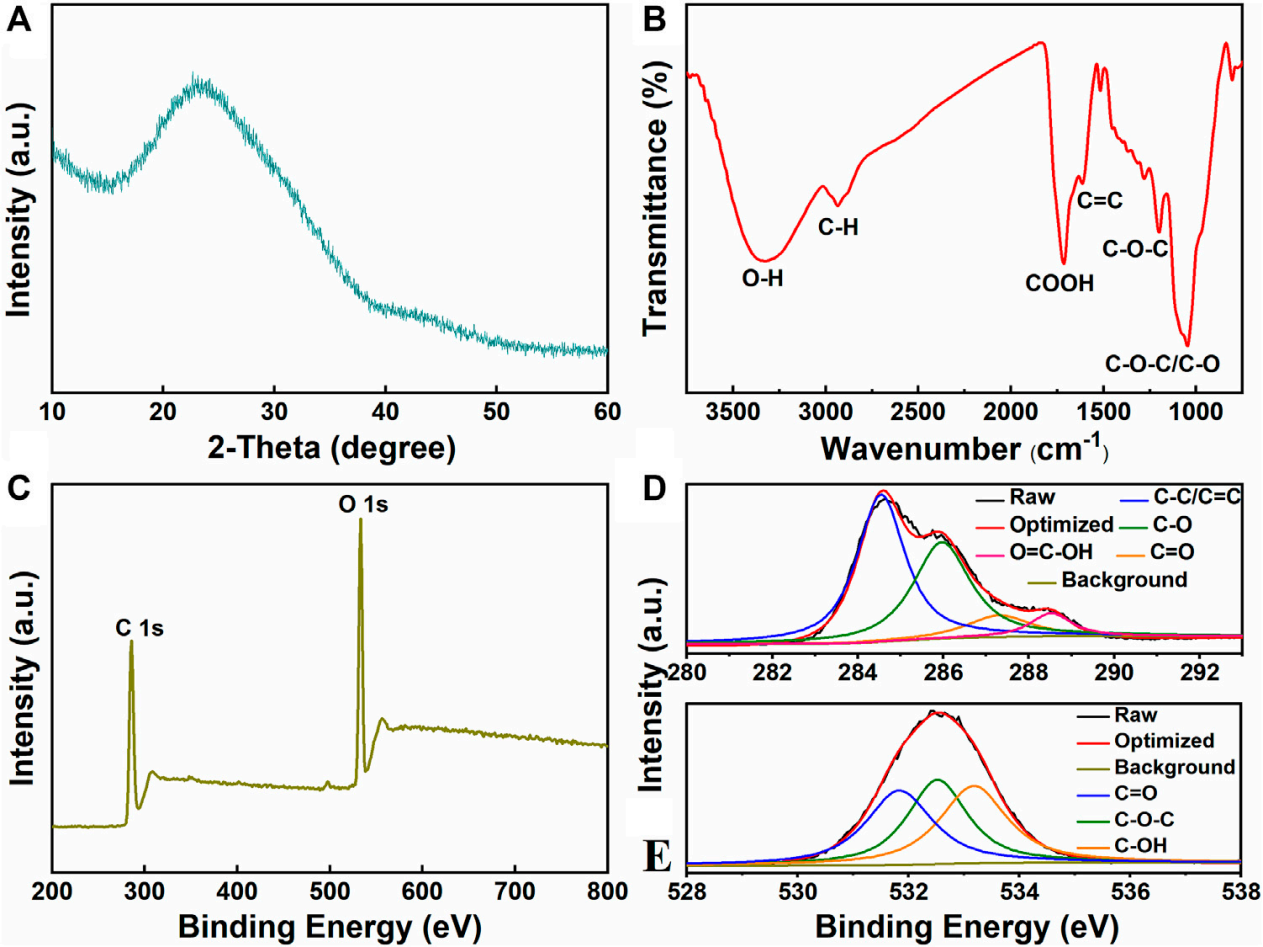
**(A)** XRD patterns, **(B)** FT-IR spectra, **(C)** XPS spectra of CQDs, **(D)** C1s, and **(E)** O1s high-resolution spectra of the CQDs.

The XRD analysis was applied to characterize the crystalline structure of CQDs. As shown in Figure 3A, the XRD pattern of CQDs has a broad diffraction peak at 23.1°, which was identical to that of a graphitic structure (Dong et al., 2012). The peak at 15.6° in the carbohydrate pulp corresponded to the amorphous structure (Supplementary Figure S4). As compared to the peak in carbohydrate pulp, the shifted peak was correlated with the crystal transition from amorphous pulp into crystalline CQDs.

The detailed functional groups in CQDs were identified by FT-IR (Figure 3B). A broad peak at 3,325 cm^−1^ is assigned to O–H stretching vibration (Ramanan et al., 2018). The peaks at 2,936 cm^−1^ and 1713 cm^−1^ are related to the stretching vibration of C–H and carboxyl (–COOH), respectively (Yang et al., 2018). In addition, the bands observed at 1,614 cm^−1^ and 1,200 cm^−1^ reveal the presence of C=C and C–O–C functional groups (Li et al., 2015). A weak signal at 1,047 cm^−1^ could be ascribed to the absorption of C–O–C and C–O. The FT-IR analysis suggested the existence of hydrophilic functional groups on the surface of CQDs. The oxygen-containing functional groups remarkably improved the stability and dispersion of CQDs in aqueous solutions (Wang et al., 2015).

To further probe the surface composition and valence state of the CQDs, XPS spectroscopy measurement was performed. The XPS survey spectrum of CQDs presents obvious bands centered at 284.2 and 531.5 eV, corresponding to C1s and O1s signals, respectively (Figure 3C). The high-resolution C1s and O1s XPS spectra of CQDs were also analyzed. The configuration of carbon was governed by the sp^2^ hybridized C–C bonds with minor existence of hydrocarbon groups and–COOH groups (Wang M. et al., 2020). As illustrated in Figure 3D, the high-resolution C1s spectrum exhibited four peaks at 284.6, 285.9, 287.4, and 288.5 eV, which are attributed to C-C/C=C, C-O, C=O and –COOH bonds, respectively (Xu et al., 2020). In the high-resolution O1s spectrum (Figure 3E), the peaks at 531.8, 532.5, and 533.2 eV are corresponding to C=O, C-O-C, and C-OH groups, respectively (Miao et al., 2018). The XPS results indicated that the prepared CQDs contained some main groups such as carbonyl and carboxyl, which were consistent with the FT-IR analysis.

### Optical Properties

**FIGURE 4:**
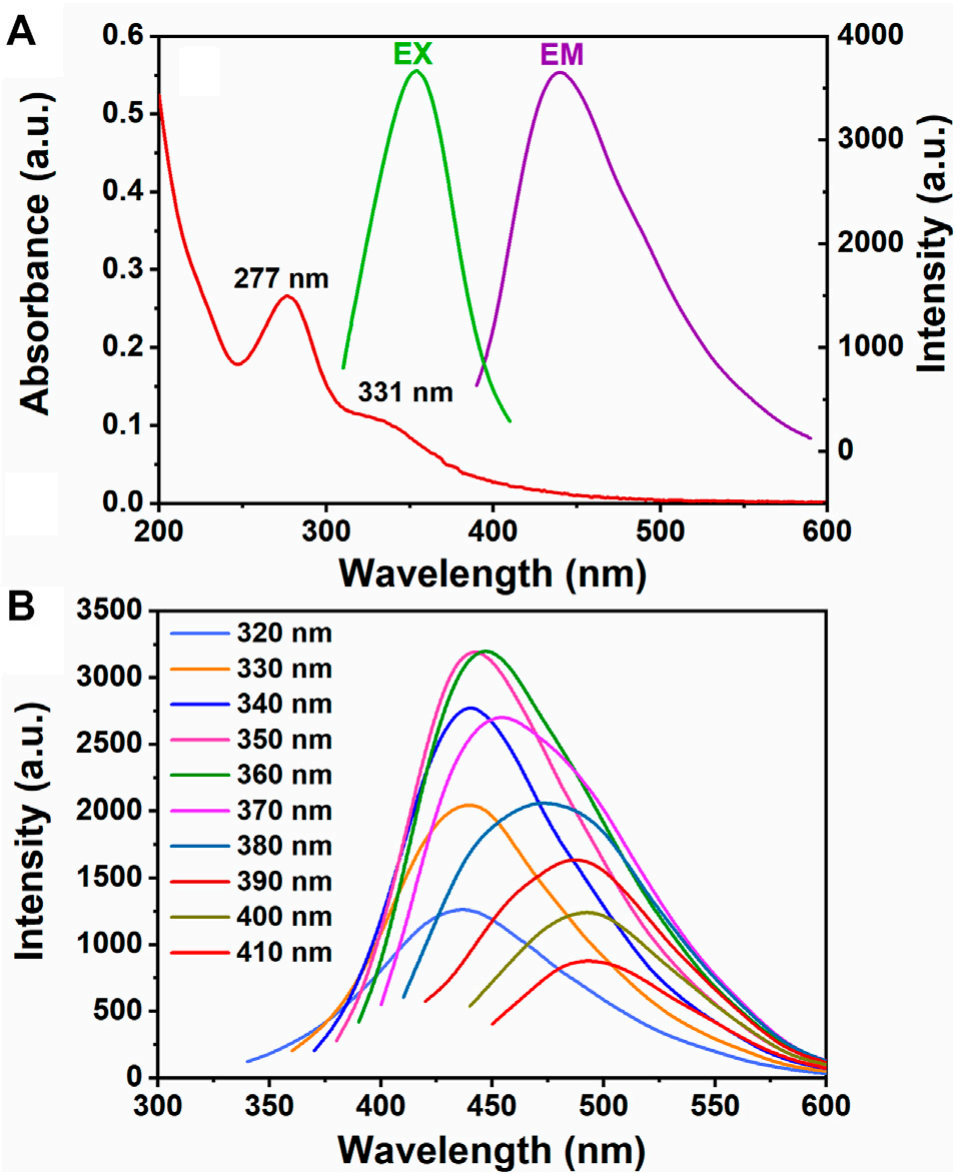
**(A)** UV–Vis absorption spectra of CQDs and the excitation/emission fluorescent spectra of CQDs. **(B)** Fluorescent emission spectra of CQDs at different range of excitation wavelengths.

The optical properties of the CQD aqueous solution were systematically studied according to fluorescence spectroscopies and UV–Vis. The CQD solution emitted bright blue fluorescence at UV illumination of 365 nm and displayed superior aqueous dispersibility. As shown in Figure 4A, the UV–Vis spectra of CQDs had a strong absorption peak at 277 nm and a weak shoulder peak at 331 nm. The peak at 277 nm is attributed to the π–π* transition of the C=C bond, in which the orbital was sp^2^-hybridized clusters (Guo et al., 2016). The peak at 331 nm is assigned to the n–π* transition of the C=O groups (Zhang and Chen, 2014). Figure 4B illustrates the fluorescence emission spectra of the CQDs under various excitation wavelengths. The emission peaks of CQDs were gradually red-shifted with the increase in excitation wavelength from 320 to 410 nm. The CQDs exhibited a maximum emission at 440 nm when the excitation wavelength was 350 nm. This result suggested that the fluorescence emission spectra of CQDs were heavily dependent on the excitation wavelength. In addition, the O-containing groups on sp^2^-hybridized carbon could induce local distortion, and the O-related defect state can further cause energy gaps. The electrons from the ground state are excited to the various energy levels of π* by excitation. The surface excited electrons are trapped by the O-related defect state, resulting in the back-transition of the electrons from the excited state to ground state (Yang et al., 2019). Therefore, the O-related defect state was an important factor for the bright blue fluorescence of the obtained CQDs.

### Detection of Fe^3+^ Ions

**FIGURE 5:**
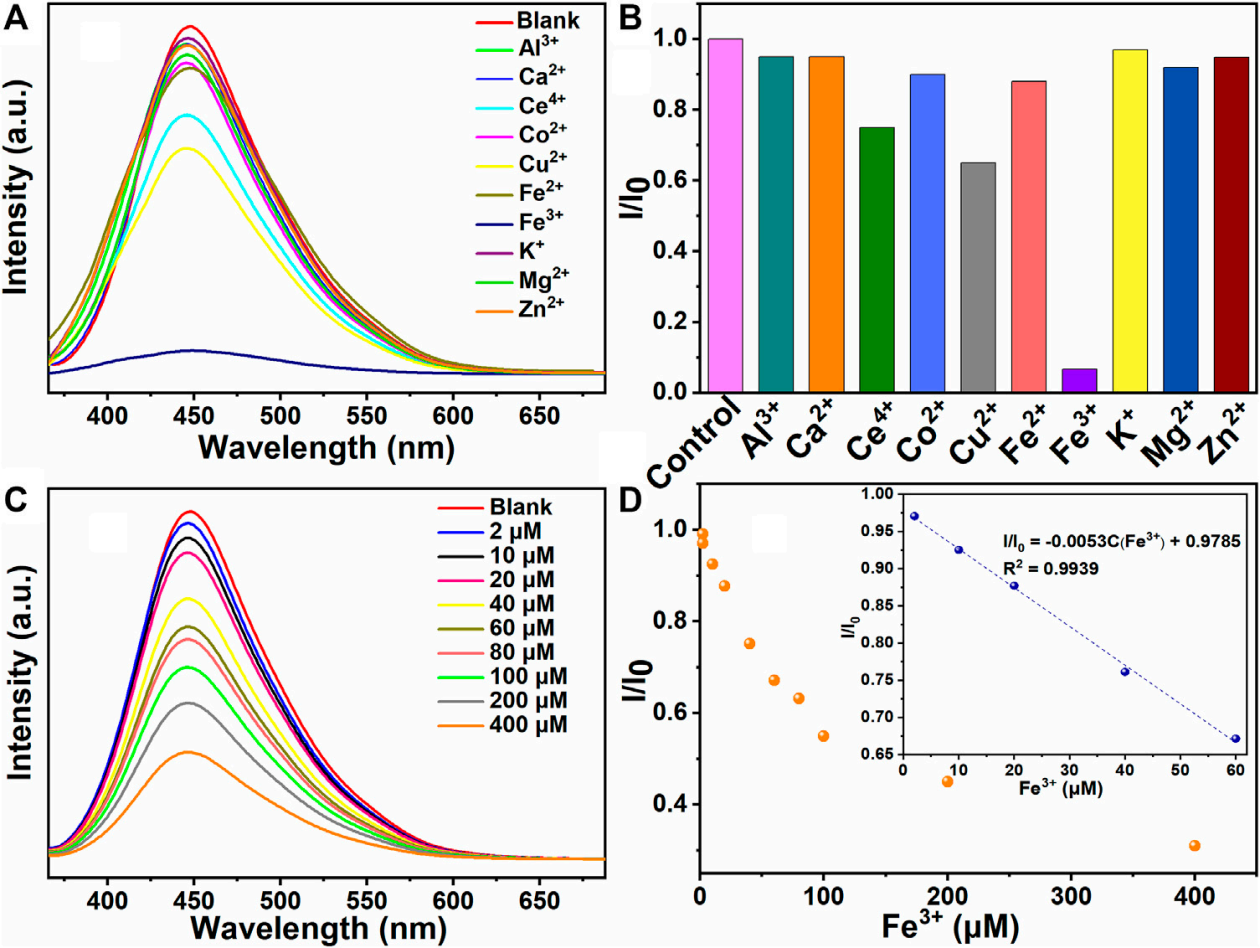
**(A)** Fluorescent emission spectra of CQDs under various metal ions. **(B)** The relative fluorescent intensities (I/I_0_) of CQDs under various metal ions. **(C)** Fluorescent emission spectra of CQDs under various Fe^3+^ ion concentrations. **(D)** The dependence of I/I_0_ value on the different Fe^3+^ ion concentrations.

In this study, CQDs with excellent fluorescence performance were successfully prepared using the solid carbohydrate pulp as precursor. The fluorescence quenching performance of the as-prepared CQDs in the presence of different metal ions was evaluated. A series of metal ions, including Al^3+^, Ca^2+^, Ce^4+^, Co^2+^, Cu^2+^, Fe^2+^, Fe^3+^, K^+^, Mg^2+^, and Zn^2+^ ions, were mixed with the CQD aqueous solution using an excitation wavelength of 350 nm for fluorescence detection. The corresponding fluorescence emission spectra are depicted in Figure 5A. The marked fluorescence quenching appeared when Fe^3+^ ions were added to the CQD solution. As shown in Figure 5B, the I/I_0_ results indicated that the Fe^3+^ ions displayed the severe fluorescence quenching capability as compared with other metal ions. It has been reported that the fluorescence quenching phenomenon was attributed to the electron transfer from oxygen-rich groups of CQDs and metal ions (Liu W. et al., 2017). Therefore, the superior fluorescent selectivity of CQDs toward Fe^3+^ ions was due to the strong coordination between Fe^3+^ ions and carboxyl groups on the surface of CQDs.

The sensitivity of Fe^3+^ ion sensing of the CQDs was carried out under various Fe^3+^ concentrations. As illustrated in Figure 5C, the fluorescence intensity of CQDs decreased with the enhancement of the Fe^3+^ ion concentrations. Varying the Fe^3+^ ion concentration from 0 to 60 μM, the variation of emission intensity (I/I_0_ value) displayed a good linear relationship with a correlation coefficient R^2^ = 0.994 (Figure 5D). The limit of detection (LOD) was calculated to be 0.978 μM based on the three times standard deviation rule (LOD = 3σ/k, *n* = 11). As shown in Table 1, the as-prepared CQDs for the detection of Fe^3+^ ions were comparable to the reported CQDs synthesized by other precursors. The presented CQDs possessed smaller size (5.24 nm), higher QY (21.7%), and wider linear range (0–60 μM) of Fe^3+^ ions as compared with the reported CQDs. The selectivity and sensitivity studies of the as-prepared CQDs suggest the promising applications of CQDs in the fluorescent sensor for the detection of Fe^3+^ ions.

**TABLE 1:** Comparison of the reported CQDs for Fe^3+^ detection.

Carbon source	Size (nm)	QY (%)	Linear range (μM)	References
Coriander leaves	1.5–2.98	6.48	0–6	Sachdev and Gopinath (2015)
Papaya	3.4	18.98	1–8	Wang et al. (2016a)
Potato	0.2–2.2	6.14	5–50	Xu et al. (2014)
Black tea	4.6	—	0.25–60	Song et al. (2017)
Egg white	2.1	64	50–250	Zhang et al. (2015)
*Prunus avium* fruit	7	13	0–100	Edison et al. (2016)
Honey	2	19.8	0–100	Yang et al. (2014)
Goose feathers	21.5	17.1	2–7	Liu et al. (2015)
Konjac flour	3.37	22	—	Teng et al. (2014)
Carbohydrate pulp	5.24	21.7	0–60	This work

### Synergistic Biorefinery Based on Catalytic Hydrogenolysis and Hydrothermal Treatment

**FIGURE 6:**
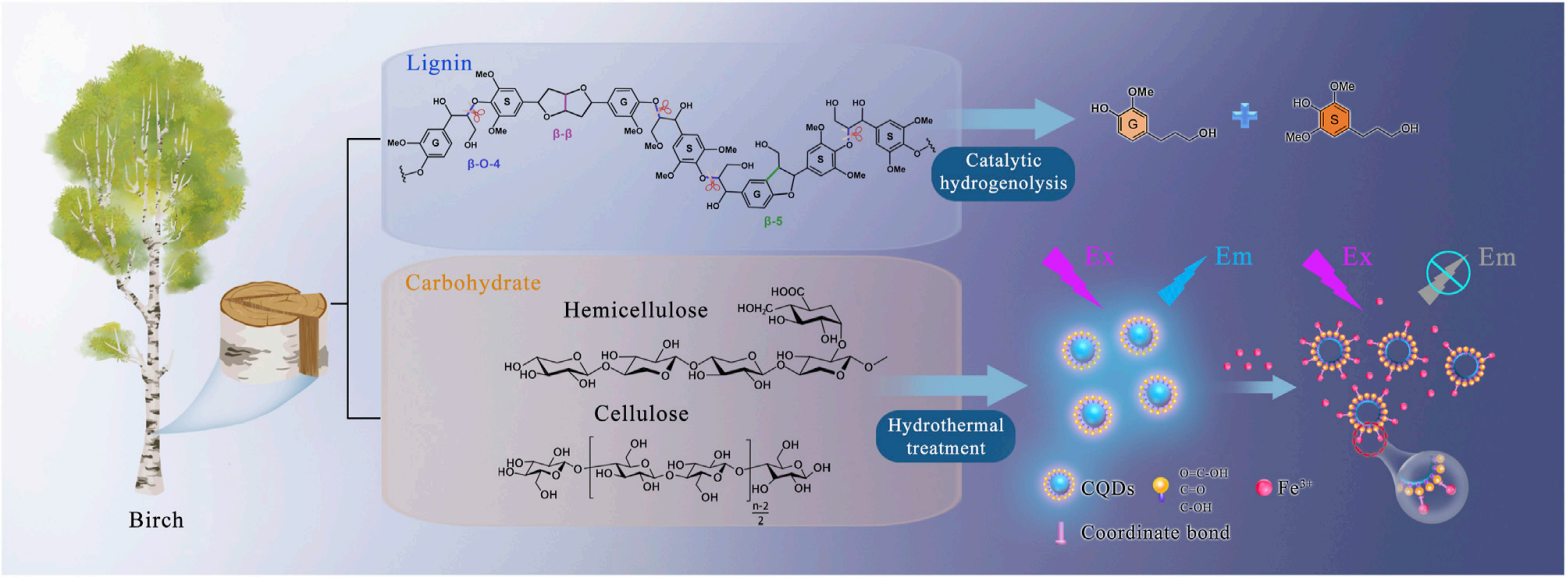
Schematic diagram for the reductive catalytic fractionation (RCF) of birch and the preparation of fluorescent CQDs.

The combination of catalytic hydrogenolysis and hydrothermal treatment opens a new direction for biorefinery configurations and synergies. The lignin portion of birch wood was first employed by Pd/C-catalyzed hydrogenolysis, while simultaneously leaving behind an essentially intact solid carbohydrate pulp that can be further processed via hydrothermal treatment. Finally, upon the treatment of birch wood (1 g) with Pd/C in methanol under 240°C for 4 h, lignin component (224 mg) can be depolymerized into monomers (100 mg), dimers, and oligomers. An available carbohydrate pulp containing cellulose (318 mg) and hemicellulose (161 mg) was further used for the synthesis of CQDs by a one-step hydrothermal process. The detailed process for transforming birch wood into phenolic monomers and CQDs is shown in Figure 6. Lignin is an amorphous and three-dimensional phenolic polymer of methoxylated phenylpropane units consisting of several types of linkages, with the most abundant being the β-*O*-4 ether linkage. Lignin in the birch wood was subjected to Pd/C-catalyzed hydrogenolysis for efficient C–O bond cleavage to obtain phenolic monomers (Parsell et al., 2013). The carbohydrates of birch were recovered and further treated for the preparation of the CQDs. The fluorescence of CQDs was quenched when Fe^3+^ ions were added into the CQDs solution. Generally, the mechanism of the fluorescence quenching phenomenon is divided into static and dynamic quenching. The observed quenching may be static quenching, resulting from the formation of a complex between the ground state of fluorescence groups and the quenching agent (Shangguan et al., 2017).

## Conclusion

In summary, we have demonstrated the integration of catalytic hydrogenolysis and hydrothermal treatment that provided a simple strategy toward the production of phenolic monomers and CQDs from lignocellulosic biomass. The efficient catalytic hydrogenolysis of birch wood with Pd/C afforded lignin monomers with a high yield of 44.6 wt%. An available carbohydrate pulp retaining 82.4% cellulose and 71.6% hemicellulose was obtained simultaneously. Hydrothermal treatment of the pulp led to the synthesis of CQDs with a yield of 21.7%. The as-prepared CQDs exhibited effective sensing potential for Fe^3+^ ions in an aqueous solution. It is anticipated that this study provides an integrated biorefinery strategy for developing new avenues for generating value-added chemicals from lignocellulosic biomass.

## Data Availability

The original contributions presented in the study are included in the article/Supplementary Material; further inquiries can be directed to the corresponding authors.
