# Supplementary material for: Integrated Cascade Biorefinery Processes to Transform Woody Biomass Into Phenolic Monomers and Carbon Quantum Dots
Source: Front Bioeng Biotechnol. 2021 Dec 23;9:803138. doi: 10.3389/fbioe.2021.803138 (PMC8733694; doi:10.3389/fbioe.2021.803138)
Supplement: Supplementary file 1 [file DataSheet1.docx]

Supplementary Material

**Integrated Cascade Biorefinery Processes to Transform Woody Biomass into Phenolic Monomers and Carbon Quantum Dots**

***Xue Chen^1^, Jiubin Zhu^1^, Wenlu Song^1^* and Ling-Ping Xiao^2,3^****

^1^*Department of Life Science and Engineering, Jining University, Jining, China*

^2^*Liaoning Key Lab of Lignocellulose Chemistry and BioMaterials, College of Light Industry and Chemical Engineering, Dalian Polytechnic University, Dalian, China*

^3^*Guangxi Key Laboratory of Clean Pulp and Papermaking and Pollution Control, College of Light Industry and Food Engineering, Guangxi University, Nanning, China*

***Corresponding Author:**

E-mail: [songwenlu0714@163.com](mailto:songwenlu0714@163.com) (W. Song), lpxiao@dlpu.edu.cn (L.-P. Xiao)

**Table of Contents**

| 1 | General Information | S2 |
| --- | --- | --- |
| 2 | Reductive catalytic fractionation (RCF) of birch sawdust | S3 |
| 3 | Chemical composition of birch sawdust and carbohydrate pulp | S4 |
| 4 | Lignin products analysis | S6 |
| 5 | Carbon quantum dots (CQDs) analysis | S7 |
| 6 | References | S9 |

# 1. General Information

**GC and GC-MS:** GC and GC-MS analyses were carried out on a Shimadzu Model 2010 plus equipped with a HP-5 column (30 m × 0.25 mm × 0.25 mm) using a flame ionization detector (FID) and a Shimadzu GCMS-QP2010SE equipped with a HP-5MS (30 m × 0.25 mm × 0.25 mm) column, respectively. The injection temperature was 250 °C. The column temperature program was: 50 °C (3 min), 8 °C/min to 280 °C (5 min). The detection temperature was 200 °C for FID.

**GPC:** GPC analyses were performed on Shimadzu LC20 series instrument via UV/vis spectroscopy at a wavelength of 254 nm, calibrated with polystyrene standards (peaks average molecular weights of 196, 580, 1320, 4830, 9970, Polymer Laboratories Ltd.). All lignin oil products were dissolved in THF (2 mg/mL) and filtered prior to injection.

# 2. Reductive catalytic fractionation (RCF) of birch sawdust

**General procedure:** Birch (1.0 g), Pd/C (100 mg, 10 wt%) and methanol (40 mL) were mixed into a 50 mL stainless steel batch reactor (Parr Instruments Co.). The reactor was sealed, flushed with N2 about three times and pressurized with 3 MPa H_2_ at room temperature. The mixture was stirred at 800 rpm and heated at desired temperature. After reaction, the reactor was cooled to room temperature and depressurized. The reaction mixture was filtered through a nylon 66 membrane filter (0.2µm), thus forming soluble (lignin oil) and insoluble fractions (carbohydrate pulp and catalyst). For the soluble fraction, the methanol was removed under vacuum, and the residue was extracted with dichloromethane and water to separate the degraded lignin (DCM phase) and sugar products (aqueous phase). The removal of dichloromethane of organic phase gave a brown “lignin oil”, which was subjected to GC-MS and GC for analyze the lignin monomers.


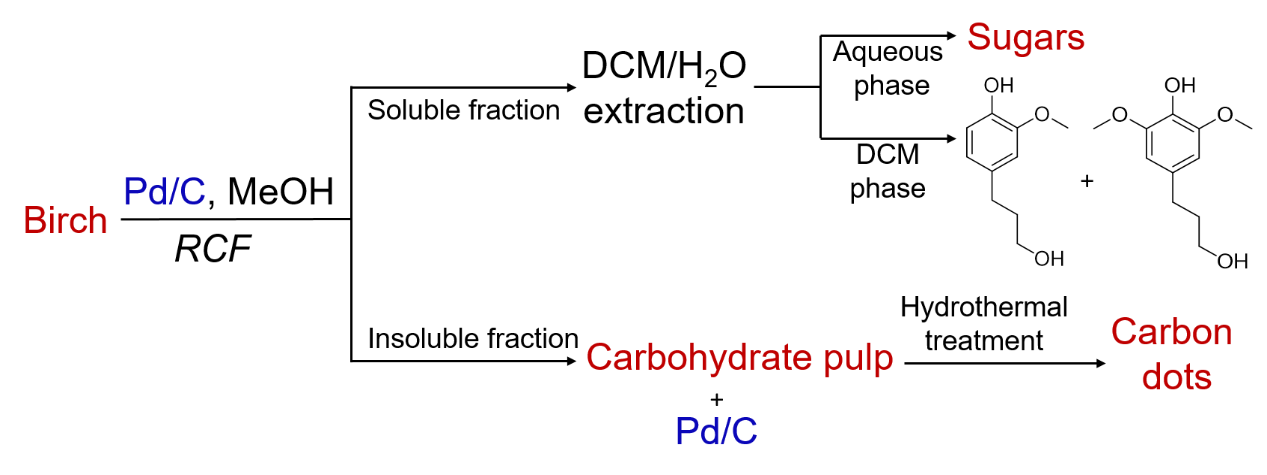


**Figure S1.** Schematic representation of Pd/C-catalyzed RCF of birch sawdust.

# 3. Chemical composition of birch and carbohydrate pulp

**3.1. General procedure**

The chemical composition was determined followed by National Renewable Energy laboratory’s (NREL) standard analytical procedure [1]. Weigh 300.0 ± 10.0 mg of the sample into a tared pressure bottle. Record the weight to the nearest 0.1 mg. Add 3.00 ± 0.01 mL (or 4.92 ± 0.01 g) of 72% sulfuric acid to each pressure bottle. Use a Teflon stir rod to mix for one minute, or until the sample is thoroughly mixed. Place the pressure bottle in a water bath set at 30 ± 3 °C and incubate the sample for 60 ± 5 minutes. Using the stir rod, stir the sample every five to ten minutes without removing the sample from the bath. Upon completion of the 60-minute hydrolysis, remove the bottles from the water bath. Dilute the acid to a 4% concentration by adding 84.00 ± 0.04 mL deionized water. This mixture was heated at 120 ^o^C for 1 h in the autoclave. After cooling, the mixture was filtered through a mixed cellulose ester (MCE) membrane filter (0.2µm).

**3.2. Analytical methods**

3.2.1 Analyze the sample for acid insoluble lignin

Vacuum filter the autoclaved hydrolysis solution through one of the previously weighed filtering crucibles. Capture the filtrate in a filtering flask. Transfer an aliquot into a sample storage bottle. This sample will be used to determine acid soluble lignin as well as carbohydrates.

Use deionized water to quantitatively transfer all remaining solids out of the pressure tube into the filtering crucible. Rinse the solids with a minimum of 50 mL fresh deionized water. Dry the crucible and acid insoluble residue at 105 ± 3 °C until a constant weight is achieved, usually a minimum of four hours. Remove the samples from the oven and cool in a desiccator. Record the weight of the crucible and dry residue to the nearest 0.1 mg.

3.2.2 Analyze the sample for acid soluble lignin

The concentration of acid soluble lignin (ASL) was determined by UV spectra by measuring the absorbance of the soluble fraction at 205 nm. Dilute the sample as necessary to bring the absorbance into the range of 0.7 – 1.0, recording the dilution. Deionized water or 4% sulfuric acid may be used to dilute the sample, but the same solvent should be used as a blank. Record the absorbance to three decimal places. Calculate the amount of acid soluble lignin present using calculation as follows:

$$\%ASL=\frac{\mathrm{UV}_{\mathrm{abs}}\times\mathrm{Volume}_{\mathrm{filtrate}}\times Dilution}{\varepsilon\times\mathrm{Weight}_{\mathrm{sample}}}*100$$

Where:

UV_abs_ = average UV-Vis absorbance for the sample at 320 nm

Volume_filtrate_ = volume of filtrate, 86.73 mL

$\varepsilon$ = Absorptivity of biomass at 205 nm

3.2.3 Analyze the sample for carbohydrates

The determination of monomeric sugars in the aqueous soluble fraction was performed on high-performance anion exchange liquid chromatography (HPAEC) system (Dionex ICS 3000, USA) equipped with a Carbopac TM PA-20 column (3 mm × 150 mm, Dionex, Sunnyvale, USA) and an amperometric detector, by comparison with authentic samples. These samples were conducted in triplicate.

Table S1. The chemical composition of birch and carbohydrate pulp generated from hydrogenolysis of birch at 240 °C for 4 h^a^

| Entry | Substrate | AIL^b^  (wt%) | ASL^c^  (wt%) | Cellulose  (wt%) | Hemicellulose  (wt%) |
| --- | --- | --- | --- | --- | --- |
| 1 | Birch | 20.1 | 2.3 | 38.6 | 22.5 |
| 2 | Carbohydrate pulp | 9.7 | 1.8 | 54.6 | 27.6 |

^a^ The chemical composition were analyzed according to the procedures of the NREL method.

^b^ AIL: acid insoluble lignin (Klason lignin).

^c^ ASL: acid soluble lignin.

# 4. Lignin products analysis

To analyze the lignin monomers after hydrogenolysis, the lignin oil and a standard (tetradecane) was solubilized in dichloromethane in a 10 mL volumetric flask. 10 μL was then analyzed on a GC (Shimadzu GC2010 series, equipped with a HP-5 column and a flame ionization detector (FID)) and GC-MS (Shimadzu GC2010 series equipped with a HP-5 MS column and a Mass Spectroscopy detector), respectively. The following operating conditions were used: injection temperature of 553 K, column heating program: 323 K (3 min), 10 K/min to 553 K, detection temperature of 563 K (for FID). The quantification of lignin monomers in the oil products were assessed by comparison with authentic samples acquired from commercial purchase or independent synthesis.


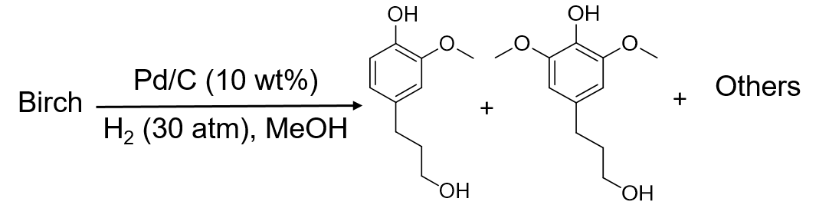


**Table S2**. Reductive catalytic fractionation of birch^a^

| The distribution and yield of phenolic monomers (wt%) ^b^ | | | | | | | |
| --- | --- | --- | --- | --- | --- | --- | --- |
| **** | **** | **** | **** | **** | Others | Total |  |
| 0.4 | 1.0 | 10.2 | 1.2 | 29.7 | 2.1 | 44.6 |  |

^a^ Reaction conditions: birch (1.0 g), 10 wt% Pd/C catalyst (100 mg), MeOH (40 mL), H_2_ (30 atm), 4 h.

^b^ Representing the monomer yield is based on lignin content in birch.

**Figure S2.** Gas chromatograms and peak identification of the lignin monomers from catalytic hydrogenolysis of birch sawdust using Pd/C catalyst at 240 °C for 4 h.

# 5. Carbon quantum dots (CQDs) analysis

Quinine sulfate with a 54% quantum yield (QY) in 0.1 M H_2_SO_4_ was used as the standard sample for the calculation of the QY [2]. CQDs were prepared using carbohydrate pulp by hydrothermal treatment at 200 ^o^C under different time period. The QY of CQDs is shown in Figure S3.

**Figure S3.** Quantum yield (QY) of CQDs obtained from carbohydrate pulp at 200 ^o^C.

**Figure S4.** The X-ray diffraction patterns of carbohydrate pulp obtained from the RCF. Reaction conditions: birch (1.0 g), 10 wt% Pd/C catalyst (100 mg), MeOH (40 mL), H_2_ (30 atm), 240 ^o^C, 4 h.

**6. References**

(1) Sluiter, A., et al., Determination of structural carbohydrates and lignin in biomass. Laboratory analytical procedure, 2008.

(2) Sahu, S., et al., Simple one-step synthesis of highly luminescent carbon dots from orange juice: application as excellent bio-imaging agents. *Chem. Commun.*, 2012, 48, 8835-8837.
